# Modeling development of breast cancer: from tumor microenvironment to preclinical applications

**DOI:** 10.3389/fphar.2024.1466017

**Published:** 2024-12-04

**Authors:** Ruizhi Tang, Xi-Qiu Liu

**Affiliations:** ^1^ Key Laboratory for Molecular Diagnosis of Hubei Province, The Central Hospital of Wuhan, Tongji Medical College, Huazhong University of Science and Technology, Wuhan, China; ^2^ School of Pharmacy, Tongji Medical College, Huazhong University of Science and Technology, Wuhan, China

**Keywords:** breast cancer, tumor microenvironment, organoids, modeling, cancer therapy

## Abstract

Breast cancer is a complex disease and its progression is related not only to tumor cells but also to its microenvironment, which can not be sufficiently reflected by the traditional monolayer cell culture manner. The novel human cancer models comprising tumor microenvironment (TME), such as tumor organoids and organs-on-a-chip, has been established in recent years to help elucidate the underlying mechanisms of tumorigenesis and promote the development of cancer therapies. In this review, we first discuss the current state of breast cancer and their treatment strategies, and elucidates the complex properties of TME of breast cancer *in vivo*. The culture models used in breast cancer research are then summarized with insights into recent development. Finally, we also conclude by discussing the current limitations and future directions of culture models in breast cancer research for providing a preclinical reference for the precise treatment of cancer patients.

## 1 Introduction

Breast cancer is the most frequently diagnosed malignancy among women all around the world (Xu et al., 2023). Thus, much effort has been focused on understanding breast cancer tumorigenesis at the molecular and cellular level for precision oncology and drug screening (Mohan et al., 2021). Although the traditional tumor monolayer cell culture model and mouse models have been critical in advancing the knowledge of breast cancer development, the success rate of the clinical development of antineoplastic drugs is much lower than that of other drugs (Guan and Huang, 2022). Breast cancer is a complex disease and its progression is related not only to tumor cells but also to their environment. The tumor microenvironment (TME) is the ecological environment on which tumor cells depend for survival and development, in which tumor cells come into contact with each other and with stroma cells as well as noncellular components. TME can significantly affect the biological properties of breast cancer cells, such as polarity, structure, resistance, migration and invasion (Bejarano et al., 2021). Therefore, the novel human breast cancer models comprising TME, such as tumor organoids and organs-on-a-chip, has been established in recent years to help elucidate the underlying mechanisms of tumorigenesis and promote the development of cancer therapies.

This review discusses the current state of breast cancer and their treatment strategies, elucidates the complex properties of TME of breast cancer *in vivo*, and summarizes the culture models used in breast cancer research with insights into tumor organoids and organs-on-a-chip. Finally, we also conclude by discussing the current limitations and future directions of culture models in breast cancer research for providing a preclinical reference for the precise treatment of cancer patients.

## 2 Breast cancer stages and current therapies

Breast cancer is a highly heterogeneous malignant tumor, and its classification and therapeutic strategies are closely related to the molecular characteristics of the tumor. Its immunohistochemical markers include estrogen/progestin (ER/PR) and human epidermal growth factor receptor 2 (HER2), and breast cancer is divided into three main subtypes: ER/PR-positive, HER two-positive, and triple-negative breast cancer (TNBC) (Harbeck and Gnant, 2017). Breast cancer has different sensitivities to different drugs because of its different molecular subtypes. Among them, ER/PR-positive breast cancers (about 70%–80%) are usually sensitive to endocrine therapy. Endocrine therapy drugs, such as tamoxifen and aromatase inhibitors, control tumor growth by blocking the estrogen signaling pathway (Barchiesi et al., 2020). HER2-positive breast cancers (about 20% of the cases) can be effectively treated with targeted therapies such as trastuzumab and pertuzumab (Al-Azawi et al., 2023; Huang et al., 2024; Lee and Park, 2021; Zhu et al., 2022). TNBC (about 10%–15%) is more challenging to treat due to the lack of these molecular markers and is usually dependent on chemotherapy (Abdou et al., 2022; Lyons, 2019). In addition to these traditional treatments, immunotherapy has also become an important part of breast cancer treatment in recent years, such as checkpoint inhibitors, which attack cancer cells by activating the patient’s own immune system (Schmid et al., 2018).

In recent years, treatment strategies for breast cancer are shifting from broad-spectrum conventional approaches to individualized and precision therapy based on molecular classification. Individualized therapy emphasizes the development of treatment regimens based on each patient’s specific tumor characteristics in order to improve treatment efficacy and reduce unwanted side effects. To achieve this goal, researchers are conducting a large number of molecular biology studies to gain a deeper understanding of the molecular mechanisms of different subtypes of breast cancer and to find new therapeutic targets (Dorling et al., 2021; Wu et al., 2021). By testing with 3D culture models that replicate the complexity of the TME, researchers can more accurately assess the effects of drug treatments *in vitro*, thus advancing the development of personalized medicine.

## 3 *In vivo* microenvironment of breast cancer

**FIGURE 1:**
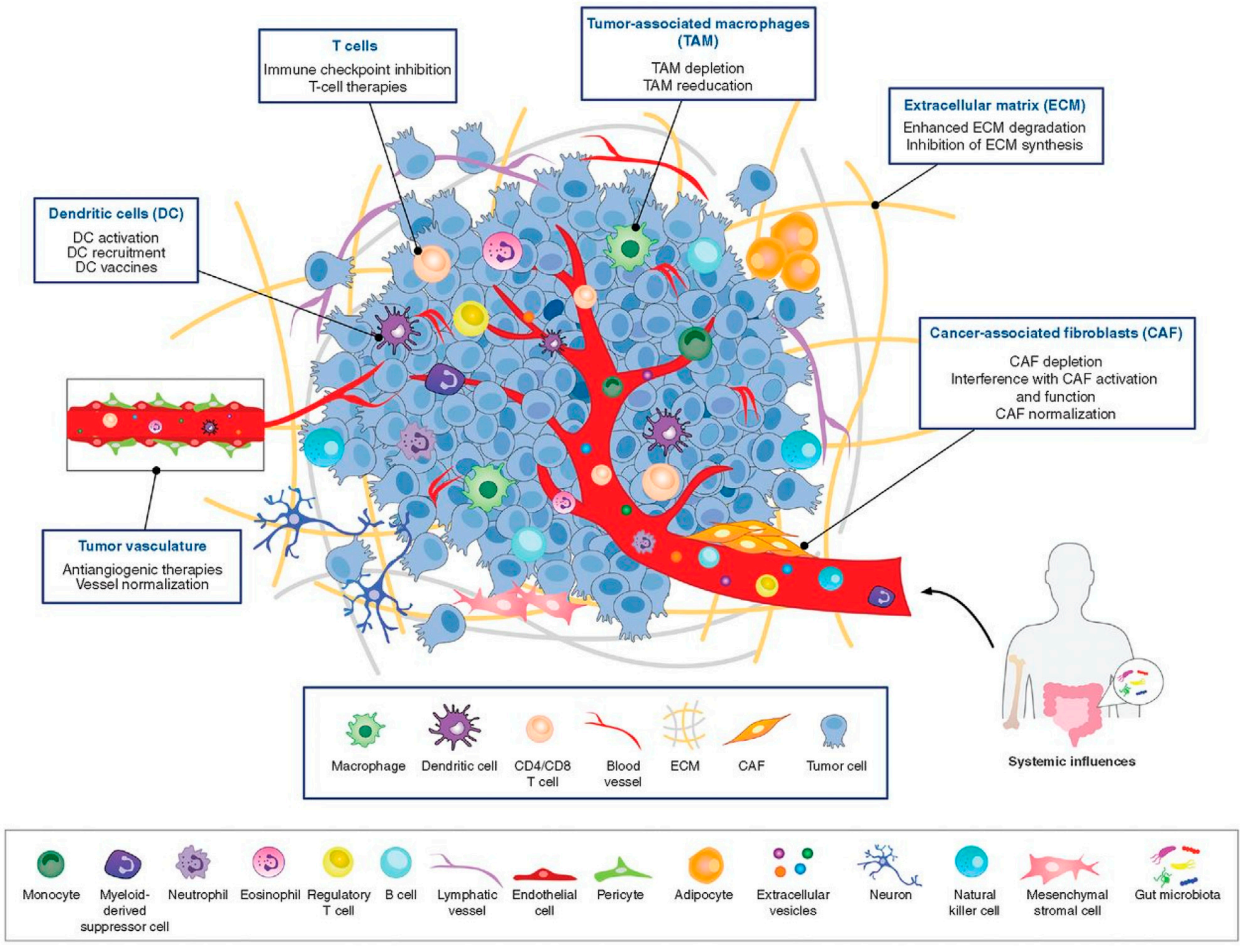
The tumor microenvironment is populated by numerous diverse cell types and ECM components (Bejarano et al., 2021).

TME plays a pivotal role in the study of breast cancer, influencing not only its occurrence and progression, but also its response to treatment. TME consists of extracellular matrix (ECM) and various cell types (Figure 1) (Bejarano et al., 2021). The ECM is a complex network of multiple proteins and polysaccharides, including collagen, elastin, fibronectin, laminin and fibrinogen (Cox, 2021; Winkler et al., 2020). These proteins not only provide structural support for tumor cells, but also influence cell behavior and signaling through interactions with cell surface receptors such as integrins (Huang et al., 2021). Remodeling of the ECM is a key process in breast cancer progression. Cancer cells and other cell types in the TME (e.g., fibroblasts and immune cells) are able to alter the composition and structure of the ECM, promoting tumor invasiveness and metastatic capacity (Goetz et al., 2011). For example, increased stiffness of the ECM can lead to enhanced activity of matrix metalloproteinases (MMPs), which promotes cancer cell migration and invasion (Kwon, 2023). In addition, the ECM plays an important role in the development of drug resistance in tumors. alterations in the ECM can affect the distribution and permeability of drugs in tumor tissues, leading to a decrease in the effectiveness of chemotherapeutic agents. At the same time, ECM can also enhance the survival and proliferation of cancer cells through the alteration of cellular signaling pathways, such as the activation of PI3K/AKT, MAPK and other signaling pathways through the integrin receptor, which in turn leads to drug resistance (Jin and Jin, 2020; Vasan et al., 2019).

In addition to cancer cells, immune cells, fibroblasts, and endothelial cells are also included in the TME. All of these cells interact dynamically with cancer cells (Hanahan, 2022; Lambert et al., 2024), and this interaction is critical for understanding tumor behavior and developing effective therapies. Below we describe several major cell types in the TME and the functions they play in breast cancer progression.

### 3.1 Tumor-associated macrophages (TAMs)

Macrophage is an important immune cell derived from blood monocytes. In the early stages of tumorigenesis, multiple signals in the TME, such as IL-6, TNF-α, and chemotactic protein-2 (CCL2), prompt monocytes to migrate to tumor tissues, where they differentiate into TAMs. The function and differentiation status of TAMs are affected by multiple signals in the TME, and depending on the microenvironment TAMs can differentiate into either pro-inflammatory (M1-type) or anti-inflammatory (M2-type) TAMs (Wang et al., 2024). M1-type TAMs have anti-tumor properties, whereas M2-type TAMs tend to promote tumor growth and metastasis (Bied et al., 2023). In breast cancer, M2-type macrophages are widely regarded as the predominant type of tumor-associated macrophages, and are also considered to be broadly defined as TAMs, which support tumor growth through the secretion of growth factors such as TGF-β, VEGF, and EGF. At the same time, TAMs promote tumor metastasis by secreting MMPs (Yu et al., 2021). In addition to their role in tumor growth and metastasis, TAMs have also been found to be closely related to drug resistance in breast cancer. Studies have shown that TAMs can exacerbate breast cancer drug resistance by promoting anti-apoptotic signaling or suppressing immune responses within tumor cells. Specifically, TAMs inhibit T-cell activation and anti-tumor immune responses by secreting a series of cytokines and signaling molecules, such as IL-10 and TGF-β, thereby promoting tumor escape and drug resistance (Ruffell and Coussens, 2015).

### 3.2 Tumor-associated fibroblasts (CAFs)

CAFs are an important type of mesenchymal cells in the TME, which are derived from normal fibroblasts or other precursor cells, such as bone marrow-derived fibroblasts, and have diverse biological functions that have an important impact on tumor development. Firstly, CAFs remodel the ECM (Chhabra and Weeraratna, 2023) of tumor tissues by secreting ECM components such as collagen and fibronectin, changing their composition and structure. This remodeling of the ECM not only increases the stiffness and stability of the tumor tissue, but also affects the interaction between tumor cells and the ECM (Erdogan et al., 2017). CAFs promote the degradation of the ECM by secreting enzymes such as MMPs, thereby increasing the possibility of invasion and metastasis of cancer cells (Chen and Song, 2019). Secondly, CAFs promote tumor cell proliferation and angiogenesis by secreting into FGF, VEGF and TGF-β (Sewell-Loftin et al., 2017). These growth factors not only directly affect the biological behavior of tumor cells, but also interact with surrounding cells and ECM to further regulate the microenvironment of TME. In addition, CAFs play an important role in regulating the response of breast cancer cells to therapy, such as reducing drug permeability by increasing the density and stiffness of the ECM, or activating anti-apoptotic and anti-drug pathways in tumor cells by secreting growth factors and cytokines to activate the signaling pathways IGF-1 and HGF in cancer cells (Dai et al., 2024; Guo et al., 2023).

### 3.3 Tumor infiltrating lymphocytes (TILs)

TILs are lymphocytes that enter tumor tissues, and they include multiple subtypes, such as CD8^+^ cytotoxic T cells, CD4^+^ helper T cells, regulatory T cells (Tregs), and B cells (Denkert et al., 2018). TILs play a key role in the tumor immune response. In particular, CD8^+^ T cells, which directly kill cancer cells, and CD4^+^ T cells, which regulate the immune response through the secretion of cytokines (Künzli and Masopust, 2023). TILs are an important component of the immune microenvironment in breast cancer, and their density and activity are strongly correlated with the biological behavior and clinical outcome of the tumor (Dieci et al., 2015). A high density of TILs is usually associated with a better prognosis and a positive response to chemotherapy and immunotherapy. Although TILs have anti-tumor potential, in some cases, such as TAMs or the microenvironment can inhibit the activity of TILs by expressing inhibitory molecules (e.g., PD-L1), leading to resistance to therapy (Acar et al., 2022; Liefaard et al., 2024).

### 3.4 Other types of cells

In TME, in addition to the cells mentioned above, a number of cells are involved in its composition, including natural killer cells (NK), dendritic cells (DC), endothelial cells and mesenchymal stem cells (MSCs). These cells also play crucial roles in the TME, and together they regulate tumor growth, metastasis, and therapeutic responses (Bejarano et al., 2021).

DC, as antigen-presenting cells, activate the immune response of T cells by phagocytosing and processing tumor-associated antigens and presenting them to T cells, and also enhance the activation and proliferation of CD4^+^ and CD8^+^ T cells by secreting pro-inflammatory cytokines such as IL-12 and TNF-α, which promote tumor-specific immune responses NK cells have a direct killing effect on tumor cells (Yin et al., 2021).

NK cells exert important anti-tumor immune activity by recognizing and killing tumor cells that express abnormal proteins or lack MHC-I molecules, and also secrete pro-inflammatory cytokines such as IFN-γ and TNF-α to activate and enhance the anti-tumor immune response of other immune cells (e.g., DCs and T cells) response (Fang et al., 2022). They help activate and enhance the immune system’s response to defend against and remove tumor cells and are expected to be an important component in breast cancer treatment (Chauhan et al., 2023).

Endothelial cells are a major component of blood vessels and are critical for tumor angiogenesis. By promoting the formation of new blood vessels, endothelia1 cells support the growth and spread of tumors by providing them with the necessary oxygen and nutrients. In addition, they may influence the metastasis and invasion of tumor cells by secreting various growth factors and cytokines (Hida, 2022). Endothelial cells may also enhance the metabolism of chemotherapeutic agents by improving the blood supply to tumors and reducing the concentration of therapeutic agents in tumor tissues (Dai et al., 2024).

MSCs are able to differentiate into a variety of cell types and promote tumor cell growth and metastasis through the secretion of cytokines and chemo-signals such as TGF-β and IL-6 (Han et al., 2022). MSCs may also increase the number or the number of immunosuppressive cells (e.g., regulatory T cells and suppressor DCs) in number or activity, decrease the activity of immune cells (e.g., NK and T cells), and regulate the levels of immunosuppressive factors produced by immune cells, thereby creating a more immune-tolerant TME. Secondly, MSCs may promote the proliferation of tumor cells and thereby increase their tolerance to treatment by secreting growth factors such as EGF and FGF-β (Han et al., 2022).

## 4 Culture models for breast cancer therapy studies

**FIGURE 2:**
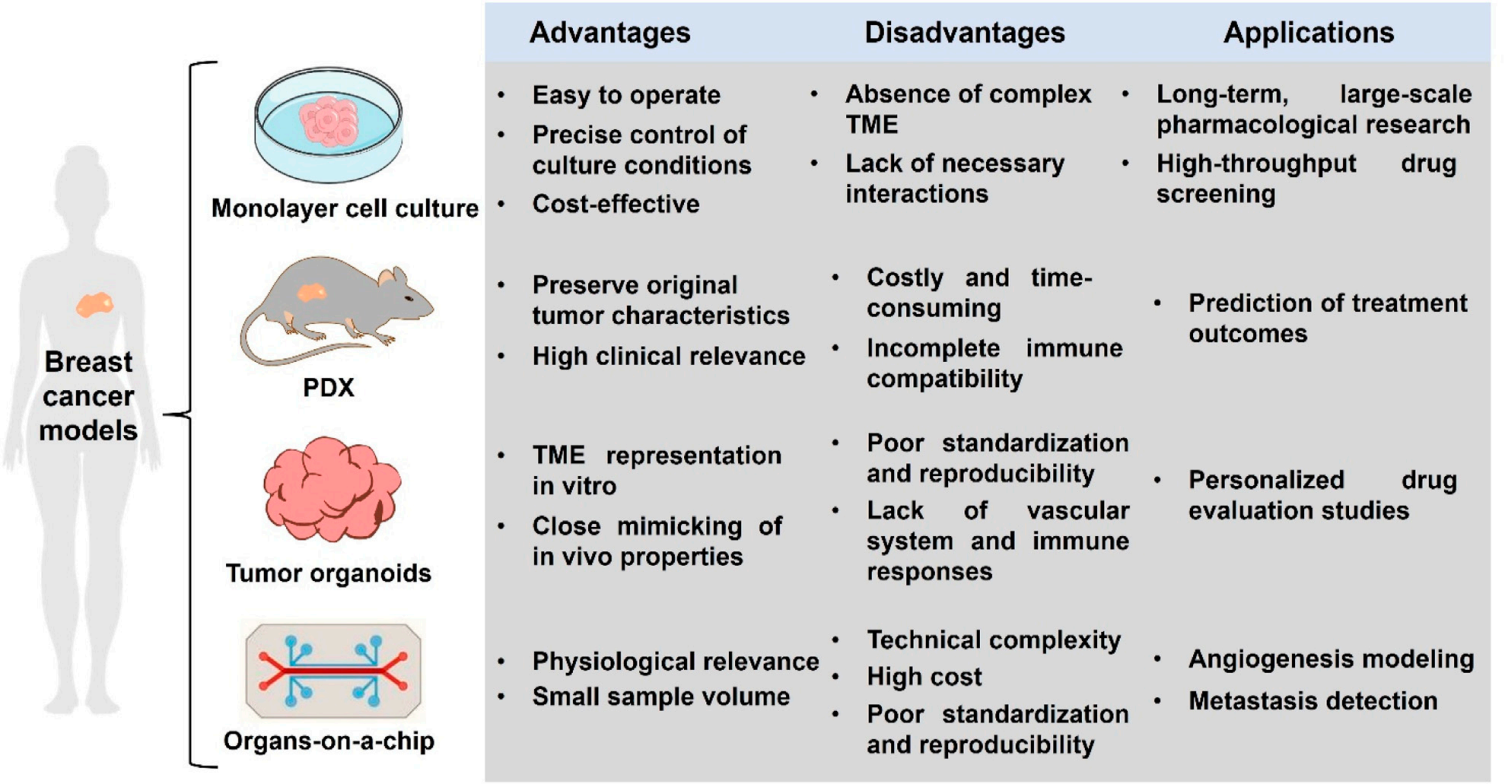
Summaries of advantages, disadvantages and of different breast cancer models.

The use of breast cancer models as a preclinical model for functional precision oncology is not new. The monolayer cell culture has been used for decades for pharmacological research and drug screening. In recent years, the advents of patient-derived xenografts (PDX), tumor organoids and organs-on-a chip have revolutionized breast cancer modeling (Figure 2).

### 4.1 Monolayer cell culture

The tumor monolayer cel1 culture model, also known as two-dimensional cell culture, is currently the most widely used culture method. The cell source for traditional monolayer cell culture can either be an established cell line, such as MCF-7, MDA-MB-231, etc., or tumor cells can be obtained from a patient’s tumor tissue. Tumor cells are inoculated into Petri dishes or culture flasks, and appropriate medium and culture conditions are provided to promote their growth and proliferation. Monolayer cell models are widely used in tumor biology and drug screening studies because they are easy to operate and allow precise control of culture conditions. However, there are some limitations, for example, the monolayer culture model cannot fully simulate the complex TME, such as cell-cell interactions, cell-substrate interactions, and vascular networks, which are microenvironmental factors that have an important impact on breast cancer growth, metastasis and drug response. Although the application of monolayer cell culture in drug screening is characterized by high throughput, the drug response results obtained *in vitro* may differ from the *in vivo* response due to the inability to mimic the complex drug metabolism and drug target environment *in vivo*, which limits the efficiency of breast cancer drug screening and drug discovery (Bahcecioglu et al., 2020). Despite the limitations of monolayer cell culture models for breast cancer, they remain one of the indispensable and important tools in biomedical research.

### 4.2 PDX

PDX is a model in which human tumor tissue is implanted directly into immunodeficient mice. The transplanted tumor cells grow progressively in the animal to form a graft that retains the histological structure and cellular heterogeneity of the primary tumor (Yoshida, 2020). PDX models are widely used in various fields of breast cancer research, including tumor pathogenesis, drug screening, and evaluation of individualized treatment options. For example, Guille et al. constructed a breast cancer PDX model and verified its proximity to the patient’s original tumor in genomic mapping, and then screened the antitumor activity of 45 compounds using this PDX model, and found that the drug activity was consistent with the *in vivo* results, and used the model to determine that birinapant may be a potential therapy for triple-negative breast cancer (Guillen et al., 2022). Elodie et al. constructed a primary breast cancer bone metastasis PDX model, and the PLK1 gene was found to upregulate in the PDX model by whole exome sequencing and gene expression analysis. Tumors in the PDX model could be shrunk by the PLK1 inhibitor. The comparison of the clinical data finally found that there was a close association between the high expression of PLK1 and the shortened metastasis-free survival and the adverse reaction to anastrozole. The results supported the clinical development of PLK1 inhibitors (Montaudon et al., 2020).

The PDX model is used as an important tool in breast cancer research, and the main advantage of this model is its ability to preserve the original tumor characteristics and its high clinical relevance (Liu et al., 2023). However, the PDX model also has some limitations. Firstly, the high cost of constructing and maintaining PDX models, as well as the long tumor growth and experimental cycles, limit the scope of their wide application. Secondly, PDX modeling requires the use of immunodeficient mice, which cannot fully reproduce the human immune response, and has a non-negligible impact on the research results (Abdolahi et al., 2022).

### 4.3 Tumor organoids

Organoids are microscopic structures organized by cells in a three-dimensional culture system *in vitro*, with the potential to mimic the microenvironment, structure and function of real organs (Lancaster and Knoblich, 2014). Originating at the beginning of the 21st century, this field has grown rapidly over the past decade with advances in stem cell technology and tissue engineering (Takebe and Wells, 2019). Initial organoid research focused on simple tissue models such as skin and intestinal models, but has since expanded to more complex organs including the heart, liver, kidney and brain (Clevers, 2016). Tumor organoids are usually composed of tumor cells as well as cells contained in the TME and are formed by specific culture conditions and biomaterial scaffolds (Ashworth and Cox, 2024; Curvello et al., 2023).

**FIGURE 3:**
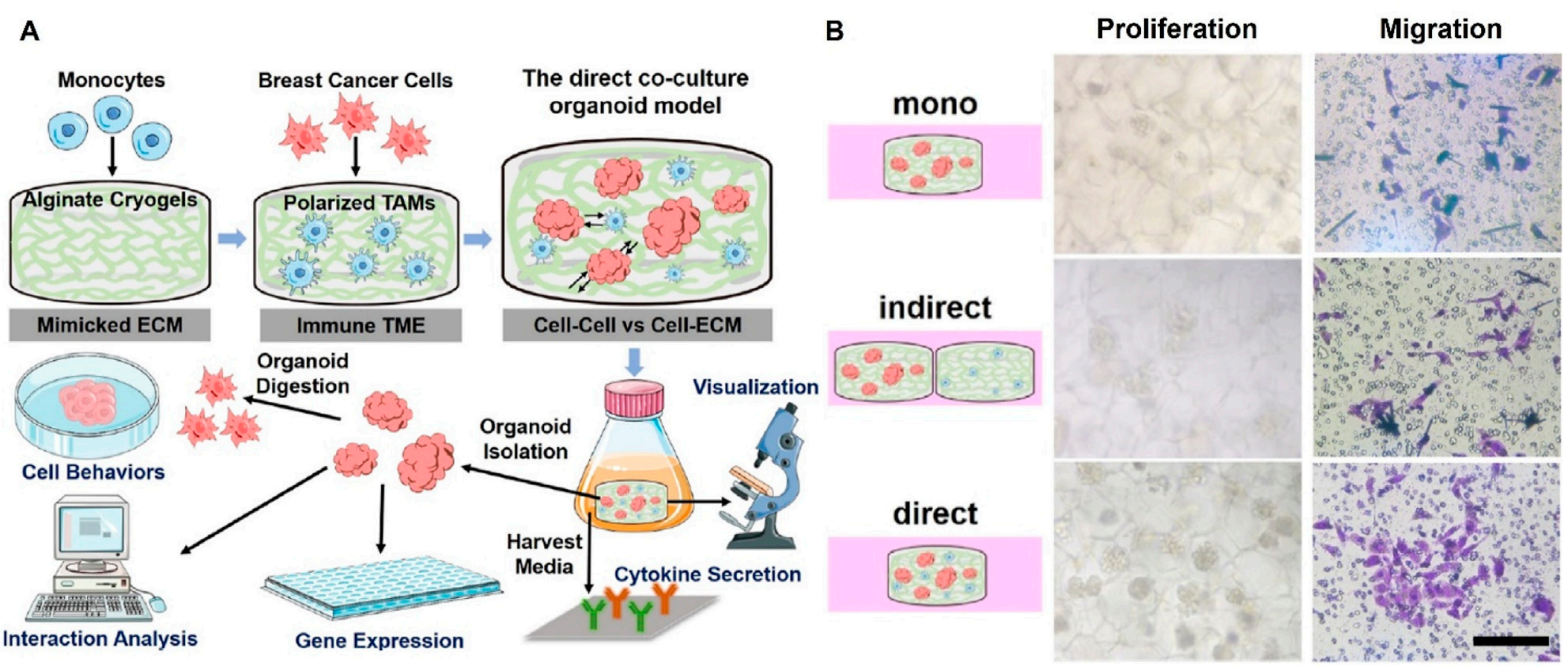
**(A)** Schematic of the direct breast cancer cells-TAMs coculture to generate immunized tumor organoids for further studies of cell behaviors, gene expressions and interactions. **(B)** Distinct proliferation and migration properties in three types of organoids formed by monoculture of only breast cancer cells and in indirect and direct breast cancer cells −TAMs cocultures (Xu et al., 2024).

Tumor organoid research is currently in a phase of rapid development, and scientists have successfully cultured multiple organoid models and used them to study disease mechanisms and conduct drug toxicity and efficacy testing. Tumor organoid technology has great potential for medical research and clinical applications, especially for those cases where it is difficult to obtain living tissue samples. Unlike traditional monolayer cell cultures, organoid models can provide a three-dimensional structure using suitable biomaterials that approximately mimic the physicochemical properties of tumor ECM in the patient. This feature is particularly beneficial for studying the intricate interactions between breast cancer cells and their surrounding microenvironment. By incorporating various components of the TME into *in vitro* culture models of breast cancer, researchers can observe and dissect the complex interactions that drive tumor progression and drug resistance (Weigelt et al., 2014). In addition, these organoid models can be extracted from individual patient tumors, capturing the unique characteristics of each patient’s cancer, which can help to study individualized responses to treatment (Bleijs et al., 2019). In the field of drug discovery, organoids can also be used for high-throughput screening, accelerating the discovery and development of new drugs (Pinto et al., 2020). Organoids have attracted much attention from researchers due to these advantages. For example, Shah et al. used sodium alginate hydrogel loaded with breast cancer cells to construct organoids, and investigated the effects of hardness and physicochemical properties of TME on the stemness of breast cancer cells by altering the physical properties of the hydrogel, which could help to develop a novel therapeutic strategy for targeting the stem cells of tumors (Shah et al., 2022). The group also constructed a breast cancer bone metastasis model to simulate the migration of cancer cells from breast cancer tissues to bone tissues by simulating the ECM of breast tissues with sodium alginate-gelatin hydrogel, and simulating the ECM of bone tissues by 3D printing bio-hybridized polycaprolactone composite scaffolds. The study demonstrated that higher hydrogel stiffness led to increased migration and invasion of breast cancer cel1s (Shah et al., 2023). Hong et al. used gelatin-sodium alginate hydrogels containing breast cancer stem cells to construct organoids for quantitative assessment of breast cancer drug resistance (Hong and Song, 2022). Xu et al. developed a type of direct breast cancer cells-TAMs coculture organoid model, in which alginate cryogels with appropriate physical and mechanical properties served as an alternative ECM. The direct coculture significantly enhanced breast cancer organoids growth and cancer aggressive phenotypes, including the stemness, migration, ECM remodeling, and cytokine secretion (Xu et al., 2024) (Figure 3). Tran et al. utilized TAMs with breast cancer cells to construct organoids for anticancer drug screening. The activation of transcription factors in terms of cytokine expression related to the TME was investigated and compared in 3D organoids and conventional 2D cell culture. The enhanced expression of STAT3, NF-kB, and p38 was only occurred in the organoids, which significantly promoted the expression of cytokines IL-6, G-CSF and GM-CSF for macrophage recruitment and polarization. The macrophages were localized inside the spheroids, whereas the breast cancer cells were located outside. However, the 2D cell co-culture showed an even distribution of the cancer cells and macrophages. Then the effects of targeting the TAMs drug PF-4136309 was tested on this model. Dead cells were found only in the center of normal culture spheroids, but they were found throughout the spheroids in the PF-4136309 treatment. The organoid model required higher drug dose and longer expose time compared to 2D culture. The phenomenon of activation of transcription factors, cytokine expression, macrophage distribution and drug resistance were masked in 2D culture (Tran et al., 2022). Abdelrahim et al. embedded breast cancer cells in a hydrogel using 3D printing and found that the antitumor drugs dihydroporphyrin E6 and tetraphenylporphyrin tetrasulfonate exhibited higher IC_50_ values in the formed organoids (Abdelrahim et al., 2022).

Organoids, a cutting-edge area of biomedical research, allow researchers to study the complexity of the human body with unprecedented depth and breadth. However, many challenges remain before widespread application, including how to ensure standardization and reproducibility of organoid models, how to increase model complexity to more accurately simulate human organs *in vitro*, and how to address bottlenecks that make it difficult to simulate the vascular system and immune responses (Wang and Fu, 2024).

### 4.4 Organs-on-a-chip

Organ-on-a-chip technology creates a microenvironment that mimics the natural growth conditions of cells by integrating living cells onto microchips composed of non-natural biomaterials such as silicon, glass, or plastic, enabling researchers to accurately study cellular behavior, cell-cell interactions, and responses to drugs and other compounds at the single or multiple cell level. Based on their application and design, organoids can be categorized into types such as single-cell analysis chips, tissue engineering chips, and drug screening chips.

**FIGURE 4:**
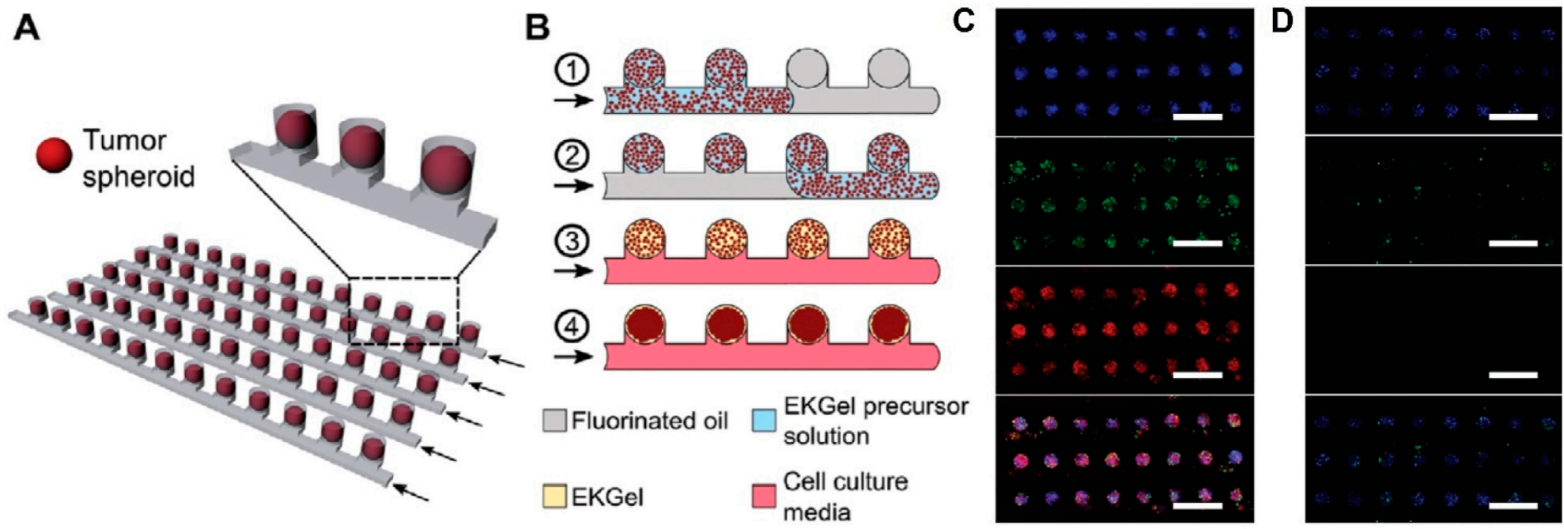
**(A)** Schematic of the microwell device comprising multiple parallel rows of tumor spheroids. **(B)** Schematic of the microwell-mediated generation of tumor spheroids. Fluorescence microscopy images of spheroids stained with live (green) and dead (red) assay after 72-h perfusion of **(C)** 10 *μ*M DOX solution and **(D)** cell culture medium (Prince et al., 2022).

Organ-on-a-chip technology has been widely used in basic biological research, drug discovery, disease modeling, and personalized medicine. Prince et al. reported a large-scale breast tumor sphere microfluidic platform using a hydrogel matrix that recapitulates the structural and biochemical properties of a fibrous breast tumor ECM, on which the drug response to doxorubicin and eribulin and clinical pharmacokinetics were simulated (Figure 4) (Prince et al., 2022). Lee et al. investigated a cardiac and breast cancer dual-organ platform introducing an immune-adaptor sensor capable of monitoring multiple biomarkers secreted by cells. Using this platform, it was demonstrated that interactions between healthy and fibrotic cardiac cells and breast cancer cells correlate with the rate of secretion of troponin T (Lee et al., 2021). Ran et al. developed a microfluidic tumor spheroids microarray model and found that its time of targeting for fluorescently-labeled liposomes was highly consistent with results from an *in vivo* mouse model. The anticancer efficacy of the monolayer cell culture model for four paclitaxel-loaded liposomes was then compared, and the results showed that the model more accurately predicted anticancer effects *in vivo*. The overall cytotoxicity using the model was significantly less than those using the 2D cell monolayer model, because the conventional cytotoxicity study was normally performed on 2D cell monolayer in a well plate where the culture medium was static. This might lead to false positive results, as sedimentation of particles may occur when the culture medium was static. Another possible reason was that spheroids possessed a dense 3D structure with tight junctions and this diffusion barrier prevented the drug-loaded liposomes to reach the center of spheroids. Finally, using this model, it was found that lower flow rate or larger tumor spheres led to less efficiency of drug absorption and poorer therapeutic effect (Ran et al., 2019).

Although organ chips face problems of technical complexity, high cost and lack of standardization and reproducibility, the application of organ chips is expected to become more widespread with the further development and improvement of technology in related fields. In the future, it is expected to become an important bridge connecting basic biology research and clinical application.

## 5 Perspectives and conclusion

Breast cancer *in vitro* culture models, an important advancement in cancer research in recent years, provide new perspectives for simulating and understanding the complexity of breast cancer. By reconstructing the three-dimensional microstructure of tumors in an *in vitro* environment, these models enable researchers to study the biological mechanisms of breast cancer, including the role of TME, cell signaling pathways, and the heterogeneity of tumor cells under conditions that are closer to the physiological state (Drost and Clevers, 2018; Dutta et al., 2017). *In vitro* culture models of breast cancer are also finding increasing applications in new drug development, drug screening, drug sensitivity testing, and design of personalized therapeutic regimens (Fröhlich, 2023).

Looking ahead, *in vitro* culture models for breast cancer will see greater development and advancement. First of all, models need to be improved to more accurately simulate TME including the interaction of tumor cells with their surrounding cells and stroma. In the future, culture conditions will continue to be improved by introducing more and more complex cell types as well as more realistic simulations of the interactions between tumor cells and their surrounding stroma. Secondly, new technologies and methods can be introduced to improve the realism and maneuverability of the models, e.g., bioprinting can be used to build more complex and fine 3D structures, microfluidics can enable drug delivery under simulated dynamic blood conditions, and gene editing can be used to build models with specific genomic variants. *In vitro* culture models of breast cancer will also play a more important role in drug development and personalized therapy. By combining the high-throughput nature of the models with bioinformatics approaches, screening of large-scale drug libraries can be realized to discover new therapeutic targets and drugs. This will provide more basis and options for the design of personalized treatment plans, making the treatment more precise and effective.

In summary, with the development of relevant techniques, *in vitro* culture models of breast cancer will continue to play a critical role in providing a platform for breast cancer treatment and research. The continued development of these models will provide an important platform and tool for our in-depth understanding of the mechanisms of breast cancer, the efficiency of drug therapy, and the impact of individual differences.
